# P-2284. "Low Incidence of Bacterial and Viral Infection after Robotic Surgery Kidney Transplant"

**DOI:** 10.1093/ofid/ofae631.2437

**Published:** 2025-01-29

**Authors:** Nikita Shah, Nicholas DiStefano, Mahmoud Morsi, Julia Bini Viotti, Jacques Simkins, Shweta Anjan, Giselle Guerra, Rodrigo Vianna, Yoichiro Natori

**Affiliations:** University of Miami Miller School of Medicine, Miami, Florida; University of Miami Miller School of Medicine, Miami, Florida; University of Miami, miami, Florida; University of Miami/ Jackson Memorial Hospital, Miami, Florida; University of Miami Miller School of Medicine and Miami Transplant Institute, Jackson Health System, Miami, Florida; University of Miami Miller School of Medicine and Miami Transplant Institute, Jackson Health System, Miami, Florida; Miami Transplant Institute, Jackson Health System, Miami, Florida; Jackson Memorial Hospital/ Miami Transplant Institute, Miami, Florida; University of Miami, Miami Transplant Institute, Jackson Health System, Miami, Florida

## Abstract

**Background:**

Kidney and kidney-pancreas transplantation should benefit patients with end-stage kidney disease and diabetes mellitus. Immunosuppressive medication are required to prevent rejection and maintain graft. However, immunosuppression increases the risk of infection. Surgical procedures also lead to inflammation post-kidney transplant, which results in opportunistic viral/fungal infections. Robotic surgery is well established to be less invasive and cause less harm in the short term for patients. However, it has never been investigated for infectious disease complications post-kidney transplantation.

**Methods:**

This was a single-center, retrospective cohort study conducted at a large kidney transplant center in the US. We included all recipients who had received kidney or kidney-pancreas transplants through robotic surgery between November 15th, 2022, and March 14th, 2023. We investigated the incidence of infection and its pathogen and the duration from transplant to infection for bacterial, fungal, and viral etiologies within 6 months post transplantation.

**Results:**

Thirty-four, and 2 kidney and kidney/pancreas transplants were performed during the study period. Thirteen (36.1%) and 4 (11.1%) of urine culture positivity (predominantly Pseudomonas and E.coli) and bacteremia were identified. The causative pathogen of 4 bacteremia includes two Staphylococcus aureus, E.coli, and Morganella. Only 6/36 (16.7%) and 3/36 (8.3%) of clinically significant CMV and BK Polyomavirus DNAemia, which required treatment, including adjusting immunosuppression, were identified. Six cases of SARS-CoV-2 and one case of influenza were also identified.

**Conclusion:**

We conducted a retrospective cohort study regarding infections after robotic kidney and kidney/pancreas transplant surgery. A very low surgical site infection and relatively low BK Polyoma viral infection rate were noted. Further study should be required to compare the incidence between robotic and traditional surgery.

**Disclosures:**

Yoichiro Natori, MD, Eurofin/Viracor: Honoraria|Nobel Pharma: Advisor/Consultant

